# Alloys as Nostrums and as Subjects for Investigation

**Published:** 1894-04

**Authors:** J. Morgan Howe

**Affiliations:** New York


### ALLOYS AS NOSTRUMS AND AS SUBJECTS FOR INVESTIGATION.[1]

BY DR. J. MORGAN HOWE, NEW YORK.

ALL will probably admit the truth of a statement that there is no reason why dentists should be ignorant of the ingredients of the materials that they use for filling teeth, as well as for the medicinal and mechanical procedures that are practised by us. We might even assert that those who work without knowledge of the constituents of their materials are not worthy to be called professional

---

[1] Read before the New York Odontological Society, January 10, 1894.

men; but then we would probably remember that the application would come so close to us as to be uncomfortable, and we would not say quite so much aloud. But in the use of alloys and cements, that are almost all of them proprietary and secret preparations, are we not certainly chargeable with being lacking in professional spirit as well as with preventing progress? We claim to be progressive, but leave the measure and direction of progress in the hands of the manufacturer; and not only in his hands, but in his head do we leave the making of our compounds for filling, so that we are ignorant of what should be added or subtracted to produce the qualities we desire.

Alloys and cements for filling are, as we use them to-day, nostrums. The makers of these wares, almost all of them, consider it to their interest to keep dentists in ignorance of their composition, but it is clearly not for *our* advantage to continue to acquiesce in this condition of affairs. Each manufacturer, like all nostrum venders, claims superior virtues for "our make," and we take and use the material thus furnished without making any claim to decide for ourselves what we desire to use.

The subject of secret preparations and their use is not a new topic in dental society meetings, but such compounds as are more used than any others have seemed to be strangely exempt from the special consideration they deserve.

In referring to the expressed opinion of our *confrères*, I cannot do better than to quote from an admirable paper by Dr. Kirk, on "The Question of Local Anæsthetic Nostrums," read before the First District Society of New York in March last. Referring to nostrums in general, he says,—

"With respect to the use of nostrums, the Code of Ethics of the American Dental Association, in Article II., Section 3, under head of 'Maintaining Professional Character,' says, 'It is unprofessional to circulate or recommend nostrums, or to perform any other similar acts,' which means that the moral sense of the better element in dentistry is against the use of these preparations. In his annual address, in 1892, the president of the Pennsylvania State Dental Society, Dr. Louis Jack, at its Cresson meeting, expressed his views upon the subject as follows: 'If we are professional at all, it is entirely inconsistent with the pretension that any one should secure control of procedures or the use of appliances for his own exclusive benefit, and unworthy of those who are under the moral obligation to fulfil the maxim, "Freely as ye have received, freely give." The endeavor to impose upon the profession by dispensing, for gain, secret formulæ of any of the preparations or materials we use is a still more reprehensible practice, and the use of such should be excluded from any and every society. To effectually stamp out this evil there appears only one means of action, which is for each practitioner to refuse to use any preparation the ingrediency and the proportions of which are unknown to him.'"

During the ensuing discussion of the address, Dr. J. Allen Osmun said, "There is no discovery or improvement in the practice of dentistry that would benefit the profession or their patients that the dentist who possesses it is not under bounden obligation to give to the profession. He owes it to them."

Under the title, "The Right and the Wrong of it," the Philadelphia *Medical News*, December 24, 1892, quotes the following from the *American Lancet:* "The *Practitioners' Monthly* says, 'It is not wrong to use a proprietary medicine because the Code says so, or because some men think so. It is wrong because it damages both medicine and pharmacy. It is wrong because it retards progress, sacrifices the good of the mass for the profit of the individual.'"

It seems strange, indeed, that, such being the convictions of representative men, and of very many others, we have continued so long using materials for filling, the ingredients of which we are ignorant of, applying professional sentiments mostly to preparations least used. Manufacturers offer their wares, making their claims of all the desirable virtues, and we accept without knowledge of the composition of the material, using and commending, perhaps, but unable to tell what ingredients and what proportions produce the qualities we approve of. Various efforts have been made to interest dentists in the composition of alloys, but without very much effect.

In 1874, Dr. S. P. Cutler presented a number of interesting facts about the amalgamation of several metals and their characteristics, and a posthumous paper on amalgam, by the late Professor T. B. Hitchcock, gave the result of much study and observation on his part and that of others. During more recent years two manufacturers (W. M. Speakman, of Philadelphia, and the Kellar Dental Company, of Indiana) have made alloys of which they have published the formulæ, but, so far as I am aware, they have not attracted much attention.

Dr. J. O. Kellar, of the Kellar Dental Company, analyzed sixty-five different popular alloys, designated by as many names, and published the formulæ in a circular advertising the non-secret preparations of the company. I am not aware of the degree of success attending the effort to introduce alloys and cements of known composition, but the analyses referred to received a certain endorsement by being embodied in the article on "Metallurgy" in the "American System of Dentistry." This list of analyses is an interesting subject for study, and, taken together with the prices of the alloys, is a serious commentary on the judgment of manufacturers regarding the ignorance or indifference of dentists as to the commercial value of the materials they use in practice. All these sixty-five claimants for favor must be pushed and advertised, and of course the dentist must pay for the latter expense as well as for the material and labor entering into the composition of the material itself. Many of them are mere duplicates of each other, differing only in their fanciful names. It seems to me that this Society could not take a more worthy or progressive step at the present time than to provide for the expense of a series of systematic experiments by a competent chemist and physicist, to determine with some definiteness what properties are conferred on alloys by the various metals used, and to decide upon some two or more formulæ that shall seem to fulfil our requirements in the greatest degree, and also to decide with more fulness questions that I have touched upon in some experiments I shall make report upon. Such a movement would be a beginning in the direction of learning to prescribe what ingredients and qualities we want. We could soon begin to use alloys with a better understanding of what the different compounds would accomplish.

There are six metals that may be said to have been commonly used in compounding alloys, as shown by the analyses referred to,—namely, tin, silver, gold, platinum, copper, and zinc. Only one maker of alloys (Dr. Chase) has used antimony, and the use of cadmium has probably been for very good reasons discontinued. Aluminum has been very little used as yet as a constituent of our alloys, but its properties in this regard ought to be investigated. Dr. Flagg has given a great deal of information regarding alloys in his book, but there are questions which he has not settled, and much that we might yet learn.

Last June, Dr. Crouse sent me samples of two alloys which he proposed to have made to sell to members of the Dental Protective Association, and I have been interested in them for that reason, and also because one of them contained aluminum. I had never used an alloy containing this metal, and it seemed to have peculiar properties, besides that which was claimed for it, that it would not discolor or tarnish in the mouth. I am unable to make any report on this latter quality, but the color and working qualities of the alloy have seemed to me very favorable, and have commended themselves to me thus far. Early last spring, Professor Mayr made some alloy for me from a formula I sent to him. In the letter from him, he expressed the opinion that coarse-cut alloy was better than fine, in requiring less mercury and in shrinking less. My preference had always been for fine-cut alloy, and as I thought probably the opinion was the result of theorizing, my mind was not very much impressed by it; but the professor expressed somewhat the same opinion when he addressed this Society last June. He said at that time, speaking of alloys for amalgam, "The coarser the grain, the less mercury is taken up; the more slowly the process of hardening, but the harder the amalgam, the less likelihood of shrinking. The finer the grain, the more rapidly it hardens, takes up more mercury, and is less likely to expand than to shrink. This matter of fineness of grain seems to me of more importance than even slight variations in the composition." The 1888 circular of the Kellar Dental Company contains a statement that coarse-grained alloys are best; that amalgam made from such particles will work stiffer in the hand, but will have less tendency to shrink because of quicker setting. They therefore advised the use of coarse-grained alloy. Nevertheless, the advertisement of this company in the January number of the INTERNATIONAL DENTAL JOURNAL says that Kellar's alloys are cut into microscopical shavings, so that they manipulate with a velvety feeling in the hands, and that the demand for amalgams with a fine plasticity is almost universal. "Hence we send the microscopic shavings instead of the coarse powder, unless otherwise ordered."

We cannot blame them for sending what is popular when we don't take the trouble to find out for ourselves what is best, and so order it.

In Dr. T. B. Hitchcock's paper above referred to, he said, "An amalgam hardens quicker when the filings or cuttings of the alloy are moderately coarse than when they are fine."

Here are contradictory statements from three parties, each of whom may be credited with considerable information in regard to alloys, but neither of them states that the opinion expressed is the result of experiment, and there is no reference to the amount of mercury to be used. A fair inference would be that the expressions regarding the relative qualities of fine- and coarse-cut alloys is meant to apply to them when mixed with the same weights of mercury. I have made a few experiments, the results of which are interesting, and also very valuable, I think.

First, I will show some bottles containing fine and coarse particles of alloys, which I was able to obtain by sifting the different alloys; some are very fine grains and some are quite coarse. But both fine and coarse of the same kind represent the two extremes out of the same lot, as furnished by the maker. The medium cuttings—constituting the greater part of the alloys as prepared—were not used for testing-purposes. I weighed a given equal number of grains of alloy of coarse-cut and of fine-cut (say twelve grains), and a given equal number of grains of mercury (say six grains) for each portion of alloy, and on making the amalgamation I found that the mass made with coarse-cut alloy was considerably more plastic than the mix made with fine-cut. So that the statement of the Kellar circular, which states that amalgam made with coarse-cut alloy "will work stiffer in the hand," is contradicted as a generalization by my experiment. The same relative result was obtained with three different alloys, showing the coarser-cut particles to make a much more plastic mass than the fine-cut, with the same relative quantity of mercury. I made other amalgamations in the same way, with a progressively diminishing amount of mercury, until the mass made with fine-cut alloy was so powdery as to be almost unworkable, while that made with the coarse cut was less powdery and more easily worked. In regard to the rapidity of hardening, I made with the same alloy two amalgamations, taking equal weights of coarse- and fine-cut metal, with the same relative weights of mercury. These were packed in short sections of glass tubing, and five minutes afterwards pushed out, so that their progressive hardening could be observed; and the fine-cut was found to set much quicker than the other. Being tested five minutes, ten minutes, an hour, an hour and a half, and two hours after, it was found still to be considerably harder, but four and five hours after packing the conditions seemed to be changed, and the coarse-cut mix was rather the hardest. This was repeated with three different alloys, and with practically the same results.

Finally, taking equal weights of coarse- and fine-cut alloy, I took an amount of mercury for the fine cuttings just sufficient to make a workable mass, and for the coarse cuttings of the same alloy I used one-third (thirty-three and one-third per cent.) less mercury. Making amalgamations under these conditions, the amount of plasticity of the two mixes was so nearly equal that no difference could be perceived. The coarse-cut mix was quite as workable, with one-third less mercury, as the fine-cut mix with the larger quantity. These masses were packed in short sections of glass tubing, and afterwards pushed out for observation. Repeated testing showed no perceptible difference in rapidity or degree of hardness between the two samples of the same alloy until two or more hours after mixing, when the plug made from coarse particles seemed to be gaining in hardness over the other. These tests were also made under the same conditions with three different alloys. The forumlæ of the alloys used differ considerably, so that it seems quite probable that these results will apply to all our alloys. One formula is stated by the maker as, silver, 56; tin, 40; gold 4. Another, Dr. Crouse has stated to be (approximately), silver, 42; tin, 50; copper, 5; aluminum, 1; zinc, 1; gold, 1; and the third, made by Professor Mayr, is, silver, 52; tin, 40; copper, 3; gold, 5.

These facts are presented with a hope that we may in some way begin investigations that shall increase our knowledge of our compound filling-materials.

In regard to alloys, I think we ought to go even further than suggested by Dr. Jack, "to refuse to use any preparation the ingrediency and the proportions of which are unknown to him," but we ought to refuse to use them unless the formula is published by being printed on the package.

By buying only of those who are willing to do this, a reform of this kind could easily be brought about.

